# Is Working More Costly than Waiting in Monkeys?

**DOI:** 10.1371/journal.pone.0048434

**Published:** 2012-11-07

**Authors:** Takafumi Minamimoto, Yukiko Hori, Barry J. Richmond

**Affiliations:** 1 Department of Molecular Neuroimaging, Molecular Imaging Center, National Institute of Radiological Sciences, Chiba, Japan; 2 Laboratory of Neuropsychology, National Institute of Mental Health, National Institutes of Health, Department of Health and Human Services, Bethesda, Maryland, United States of America; 3 PRESTO, Japan Science and Technology Agency (JST), Saitama, Japan; Brain and Spine Institute (ICM), France

## Abstract

We studied how value for instrumental action is discounted by predicted effort and delay. The monkeys were trained to perform instrumental trials that required a bar release when a visual target changed from red-to-green. There were two trial conditions. In delay trials, after the monkeys performed one instrumental trial correctly a reward was delivered 0–7 seconds later. In work trials, the monkeys had to perform 0, 1, or 2 additional instrumental trials to obtain a reward. The lengths of trials in delay matched the time it took to complete work trials. The length of delay or number of trials was indicated by a visual cue presented throughout the trial. Our hypothesis was that the monkeys would all show temporal discounting of reward in the delay trials, and that in the work trials the monkeys’ performance might reflect an additional cost due to working. The error rate increased linearly as remaining cost increased for all 8 monkeys. For 4 monkeys the error rate was significantly larger in work trials than in delay trials (effort sensitive monkeys). For the other 4 monkeys there was no significant difference in error rate (effort insensitive monkeys). Since the error rate has an inverse relation with value for action, these results suggest that value is discounted hyperbolically by effort as well as by delay. Error rates generally increased as the testing sessions progressed and the total reward accumulated (i.e., effect of reward devaluation). The relative impact of delay and effort on error rates was reasonably stable within subjects. Thus, within the monkey population there seems to be a significant dichotomy in the sensitivity governing whether working is more costly than waiting, possibly arising from a constitutional or genetic trait.

## Introduction

Studies in economics, psychology and behavioral ecology show that the performance of rewarded tasks is affected by the anticipated or predicted physical and/or mental cost, of obtaining the reward. Delays-to-reward, effort and risk are among the types of costs that have been studied. For example, when offered a choice between a small reward that is available sooner and a larger reward that is available in the more distant future, rats [1], pigeons [2], and humans [3] frequently choose the smaller reward in such a situation. In these studies, the choice can be predicted by discounting the reward’s intrinsic value by the duration of the expected delay, an effect designated as “delay discounting” [1], [4]. Discounting of the reward value also occurs in proportion to the predicted perceived effort needed to obtain the rewards; this effect is called “effort discounting” [5].

In choice paradigms, the behavior (choice preference) reflects both the decision-making process and an upstream representation of reward value [6]. There are data suggesting it is possible to eliminate the choice process by presenting only one outcome, and measuring the performance. For example when reward is delayed, reaction times are slowed [7], [8], or, as we saw recently, the success rate in performing simple instrumental trials decreases [9]. In Minamimoto et al (2009), we estimated the motivational value for instrumental action that reflects the discounted reward value by expected delay-to-reward duration. This can be extended to discounting by workloads; when monkeys have to perform a sequence of simple instrumental trials (a reward schedule) the reward value is discounted as a function of the number of trials remaining before reward [10].

Here we ask how the prediction of upcoming workload reduces motivational value for instrumental action. The monkeys performed two trials types, one in which a visual cue indicates how long after correctly completing a simple sequential color discrimination trial, the monkey will be rewarded (trials in postponement task; [9]), and in the other the monkey performed schedules of 1–3 trials of the same instrumental task used in the postponement task (trials in reward schedule task; [11]). The time to reward was matched in the two tasks, allowing us to untangle mental cost (i.e., time consumed for performance) and physical cost (i.e., effort) from estimated workloads. We first examined whether effort discounting is separable from temporal discounting. We also assessed the form of discount of motivational value by effort. Since motivation of instrumental action is affected by subjects’ internal state, that is, changing motivational value according to satiation level, we also modeled the effort and delay discount of motivational valuation including the effect of internal drive shift (e.g. from thirst to satiate).

## Materials and Methods

### Subjects

Eight adult (5–11 kg) rhesus monkeys were used in this study. All the experimental procedures were carried out in accordance with the Guide for the Care and Use of Laboratory Animals (National Research Council 1996) and approved by the Animal Care and Use Committee of the National Institute of Mental Health and by the Animal Care and Use Committee of the National Institute of Radiological Sciences (Permit Number: 09-1035). The monkeys were kept in individual primate cages in an air-conditioned room where food was always available. Throughout the study, the animals were monitored daily by an animal research technician or veterinary technician for evidence of disease or injury (e.g., dehydration, inappetance, dehydration, diarrhea, etc.) and body weight was documented weekly. Supplementary fruit were provided daily.

### Behavioral Tasks and Testing Procedures

**Figure 1:**
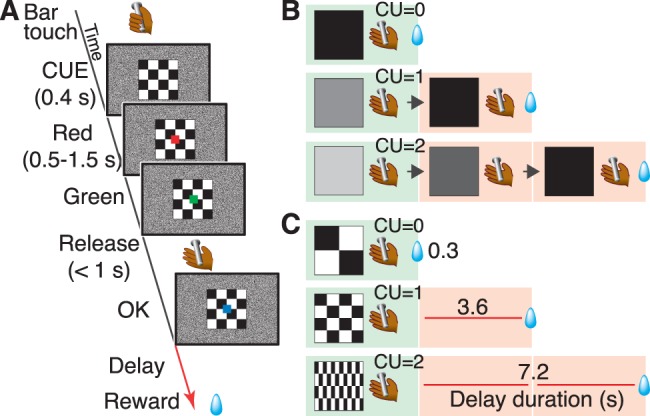
Behavioral paradigm. A Sequence of events during a trial of the work/delay task. A monkey initiated a trial by touching the bar in the chair, 100 ms later a visual cue (13° on a side), which will be described below, was presented at the centre of the monitor. After 500 ms, a red target (0.5° on a side) also appeared at the centre of the monitor. After a variable interval of 500–1,500 ms, the target turned green, indicating that the monkey could release the bar for correct performance. If the monkey responded between 200 and 1,000 ms, the target turned blue indicating the trial had been completed correctly. On correct trials, one drop of water reward was delivered immediately or after a delay (3.6 s or 7.2 s). An inter-trial interval (ITI) of 1 s was enforced before the next trial could begin. If the monkey made an error by releasing the bar before the green target or within 200 ms after the green target appeared or failed to respond within 1 s after the green target, all visual stimuli disappeared, the trial was terminated immediately, and, after the 1-s ITI, the trial was repeated. **B** Relationships between visual cues and trial schedule in the work trials. **C** Relationship between visual cues and delay duration in the delay trials. Trials on green boxed were used for the analysis. CU denotes the remaining cost (arbitral unit) to get reward, that is, either remaining workload to perform trial(s) or remaining delay periods (equivalent to a trial). It corresponds the number of red boxes placed to the reward.

For all behavioral training and testing, each monkey sat in a primate chair inside a sound-attenuated dark room. Visual stimuli were presented on a computer video monitor in front of the monkey. Behavioral control and data acquisition were performed using the REX program. Neurobehavioral Systems Presentation software was used to display visual stimuli (Neurobehavioral Systems). The basic task consisted of a series of color discrimination trials (see Fig. 1A). Each trial was initiated when the monkey touched a bar mounted at the front of the chair. To perform a trial correctly, the monkey was required to release the bar between 200 and 1000 ms after a red spot (wait signal) turned green (go signal). On correctly performed trials, the spot then turned blue (correct signal). A visual cue was presented at the beginning of each color discrimination trial (500 ms before the red spot appearing) (see Fig. 1A).

In this study, we used the work/delay task, which contained work trials (Fig. 1B) and delay trials (Fig. 1C). In work trials, the monkeys had to perform 0, 1, or 2 additional instrumental trials to obtain a reward. Work trials were the same in the multitrial reward schedule task [11], in which a visual cue indicated how many trials remaining to reward delivery. In delay trials, the monkeys performed one color discrimination trial (described above); there trials were the same as used previously in the reward delay task [9]. The visual cue indicated the combination of the trial type and requirement to obtain a reward. Pattern cues indicated the delay trials with the timing of reward delivery after correct performance; either immediate (0.3 s, 0.2–0.4 s), short delay (3.6 s, 3.0–4.2 s), or long delay (7.2 s, 6.0–8.4 s)(mean, range)(Fig. 1B). Grayscale cues indicated the work trials with the number of trials the monkey would have to perform to obtain a reward (Fig. 1C). We set the delay durations to be equivalent to the duration for 1 or 2 trials color discrimination trials, so that we can directly compare the cost of the 1 arbitrary unit (cost unit; CU).

In all cases, the visual cue provided valid information about the predicted trial outcome. This information was provided in every trial, but monkeys were not specifically trained or required to use cue-related information to perform the task. After an error (a mistimed bar release), the monkey had to repeat and correctly complete the same trial type to receive a reward.

Before entering this experiment, all monkeys had been trained to perform color discrimination trials in the cued multi-trial reward schedule task for more than 3 months. The monkeys were tested with the work/delay task as training session for 1–2 daily sessions to become familiar with the cueing condition. We tested 10–20 daily sessions. Each session continued until the monkey would no longer initiate a new trial. There were a few sessions in which the monkeys were not working well, i.e., the number of correct trials was <75% the average across sessions. These were sessions in which the monkeys did not seem motivated to work; the data from these sessions were not included in the data analysis.

### Data Analysis

All data and statistical analysis were performed using the R statistical computing environment [12]. The average error rate for each trial type was calculated for each daily session, with the error rates in each trial type being defined as the number of error trials divided by the total number of trials of that given type. A trial was considered an error trial if the monkey released the bar either before or within 200 ms after the appearance of the green target (early release) or failed to respond within 1 s after the green target (non-release). We did not distinguish between the two types of errors and used their sum except for the error pattern analysis. We used repeated-measures ANOVA to test the effect of cost type and remaining cost on error rate, on error pattern, on reaction time, and on movements during the delay.

We used the error rates to estimate the level of motivation, because the error rates of these tasks (*E*) are inversely related to the value for action (*V*)(*E* ∝ *1/V* [9]). We previously modeled the joint effect of predicted reward size and delay-to-reward on motivation with hyperbolic and exponential discounting, respectively, as follows,
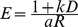
(1)

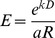
(2)where *E* is error rate, *R* is the reward size, *D* is the delay-to-reward duration, *k* is the temporal discounting factor and *a* is a constant [9]. Since we did not manipulate reward size in this study, we use simplified models as follows,
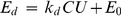
(3)

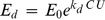
(4)where *E_d_* and *k_d_* are the error rates and delay cost factor in delay trial, *CU* is the number of remaining cost units and *E_0_* is the intercept. Eqs, 3 and 4 correspond to hyperbolic and exponential discounting, respectively. To estimate effect of remaining workloads on error rate, we used the following models,
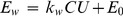
(5)

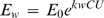
(6)


We simultaneously fitted a pair of linear models (i.e., hyperbolic discounting; Eqs, 3 and 5) or exponential models (i.e., exponential discounting; Eqs, 4 and 6) to the data by sum-of-squares minimization without weighting. The coefficient of determination (*R^2^*) is reported as a measure of goodness of fit.

To describe the effects of satiation, we divided the sessions into quartiles based on normalized cumulative reward, NCR [9]; it was 0.125, 0.375, 0.625, and 0.875 for 1st to 4th quartiles, respectively. We further describe the effect of satiation on group differences by fitting the data obtained from each monkey and average data with the functions:
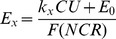
(7)where *E_x_* and *k_x_* are the error rates and cost factor in trial *x* (either work or delay trial), *CU* is the number of remaining cost units, *E_0_* is the intercept, and *F(NCR)* is the devaluation function by NCR. Previously we used a sigmoidal function as devaluation [9], [13]. Instead of a sigmoidal function, we used an exponential decay function in this study, since the error rate changed exponentially as NCR increased for all monkeys.
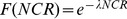
(8)where λ is a constant (the decay rate). To compare the goodness fit of discounting models with different number of parameters (i.e., Eq. 7), we used the Bayesian information criterion (BIC; BIC = −2×log-likelihood+klogN, where k is the number of free parameters and N is the number of data points) to compare the goodness of fit in each model.

## Results

### Effect of Predicted Costs on Error Rates

Eight monkeys performed the work/delay task for 10–20 sessions. The monkeys had experienced the time from release to reward delivery in the delay trial (1 remaining cost unit (CU), 3.66±0.01 s; 2 CU, 7.25±0.02 s) and in the work trials (1 CU, 3.74±0.06 s; 2 CU, 7.16±0.16 s), respectively. Since there was no significant difference in the times between two trial types (repeated measures two-way ANOVA across monkeys for trial type×remaining cost unit, main effect of trial type, F_(1, 31)_ = 0.08, *p* = 0.8), we can directly compare the effect between work and delay trials with respect to remaining cost units.

**Figure 2:**
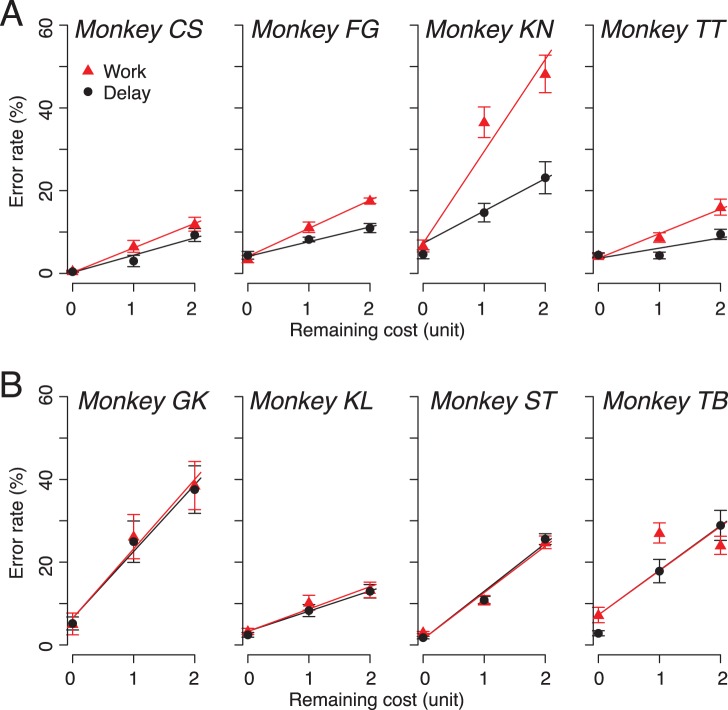
Error rates (mean ± SEM) as a function of remaining costs. **A** Effort-sensitive monkeys. **B** Effort-insensitive monkeys. Red triangles and black circles are work and delay trials, respectively. Black and grey lines are best fit lines for Eqs. 3 and 5, respectively.

We report error rates as the behavioral measure of motivational value [9]. Errors consisted of the sum of anticipatory bar release and non-release of the bar. The error rates in both work and delay trials linearly increased as remaining cost unit increased in all monkeys (Fig. 2). We performed a repeated measures two-way ANOVA across sessions for cost type×remaining cost unit on each monkey’s data. There were significant main effects of remaining cost unit for all monkeys (*p*<10^−4^). There were significant main effects of cost factor for 4 of 8 monkeys (*p*<0.05). For these 4, the error rates in work trials were significantly higher than those in delay trials (‘effort-sensitive’ monkeys, Fig. 2A). For other 4, there was no significant effect of cost type on error rates (‘effort-insensitive’ monkeys, Fig. 2B).

We estimated the cost effects on the error rates using two linear regression models (Eqs. 3 and 5, see Methods). We summarize the best-fit free parameters for effort sensitive and insensitive monkeys in Tables 1 and 2, respectively. As shown in Figure 2, error rates were well explained by Eq. 3 and 5 (average *R^2^*>0.9). In effort-sensitive monkeys, the cost factor of workload (*k_w_*) was significantly greater than that of delay (*k_d_*) (*p*<0.05, paired t-test; Table 1). The extra cost in work trials (i.e., *k_w_–k_d_*) can be regarded as the proper cost of effort. There was no statistical significant difference between the two cost factors in effort-insensitive monkeys (*p* = 0.5, paired t-test; Table 2) suggesting that no extra cost was added to the cost for waiting-for-rewards in work trials. There was no significant difference in cost factors across groups (*p* = 0.2, t-test; Table 1 vs. 2).

**Table 1:** Parameters and coefficient of determination for the best fits of Eqs. 3 and 5 to the data of effort-sensitive monkeys.

Monkey	*k_w_*	*k_d_*	*E_0_*	*R^2^*
CS	5.9	4.2	0.2	0.98
FG	6.8	3.6	4.1	0.99
KN	22.2	7.8	7.3	0.95
TT	5.9	2.5	3.7	0.94
Average	10.2±8.0	4.5±2.3	3.8±2.9	0.97±0.02

The fits corresponding to these parameters are plotted in Figure 2A. Average reports mean ± SD. There is significant difference between *k_w_* and *k_d_* (*p*<0.05, paired t-test with logarithmic transformation).

Exponential models (Eqs. 4 and 6) were also fit to the data. These models fit less well for 7 of 8 monkeys (linear *R^2^* = 0.95±0.07; exponential *R^2^* = 0.82±0.14; mean ± SD, *p*<0.01, paired t-test). Thus, the error rate in this task is generally explained well by models that assume the value is hyperbolically discounted by delay and workloads.

**Table 2:** Parameters and coefficient of determination for the best fits of Eqs. 3 and 5 to the data of effort-insensitive monkeys.

Monkey	*k_w_*	*k_d_*	*E_0_*	*R^2^*
GK	16.8	16.1	6.5	0.98
KL	5.4	4.9	3.3	0.97
ST	11.2	11.6	1.4	0.98
TB	10.7	10.8	7.3	0.79
Average	11.0±4.7	10.8±4.6	4.6±2.7	0.93±0.09

The fits corresponding to these parameters are plotted in Figure 2B. Average reports mean ± SD.

### Effect of Predicted Costs on Error Pattern

To examine whether the error pattern, i.e., early or non-release, was different between cost factors, we performed a repeated measures two-way ANOVA across sessions for remaining cost unit×cost type on each monkeys’ error pattern, i.e., percentage of early errors of total. No monkey showed a significant main effect of cost type on error pattern (*p*>0.05). In 4 monkeys (*monkeys GK, FG, KN, and TT*), there was significant main effect of remaining cost units on error patterns (*p*<0.001); the percentage of early errors increased as the remaining cost decreased. There was no systematic difference in the percentage of early errors between the cost-sensitive and cost insensitive monkeys (repeated measure two way ANOVA, main effect of group, *F*_(1, 5)_ = 2.0, *p* = 0.2).

### Effect of Predicted Costs on Reaction Time

**Figure 3:**
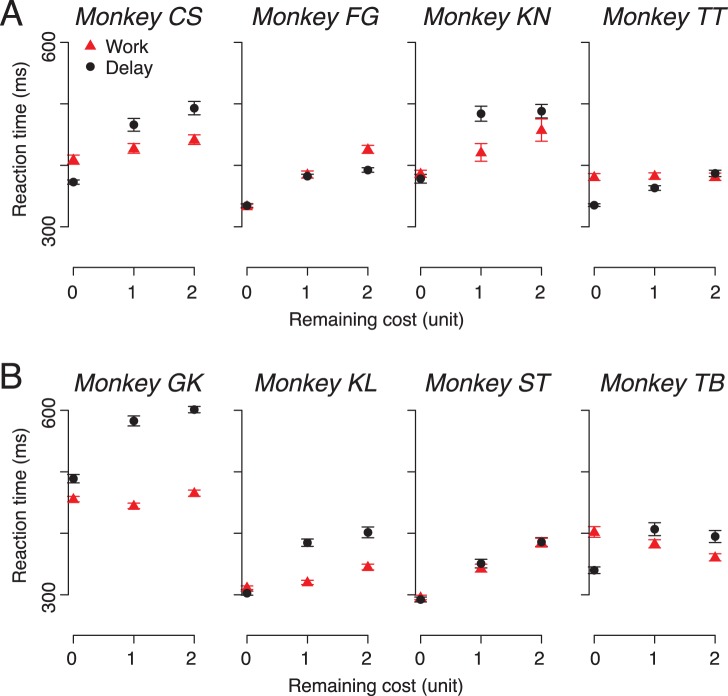
Reaction time (mean ± SEM) as a function of remaining costs. **A** Effort-sensitive monkeys. **B** Effort-insensitive monkeys. Red triangles and black circles are work and delay trials, respectively.

The reaction time increased as remaining cost increased (Fig. 3). We performed a repeated measures two-way ANOVA (remaining cost and cost type as the variables) on each monkeys’ reaction time. There was a significant main effect of remaining cost on reaction times in all 8 monkeys (*p*<10^−4^). There was also a significant main effect of cost type on reaction time in 6 of 8 monkeys (*p*<0.05). There was a significant interaction on reaction time in all 8 monkeys (*p*<0.05). There was no systematic difference in reaction time between the cost-sensitive and cost insensitive monkeys (repeated measure two way ANOVA, main effect of group, *F*_(1, 5)_ = 3.8, *p* = 0.1, Fig. 3A vs. 3B). As a consequence, there was no difference in the duration from 1st release to reward delivery in work trials between 2 groups of monkeys (1 CU, *p* = 0.8; 2 CU, *p* = 0.9, t-test).

### Incompetent Actions During the Delay Periods

**Figure 4:**
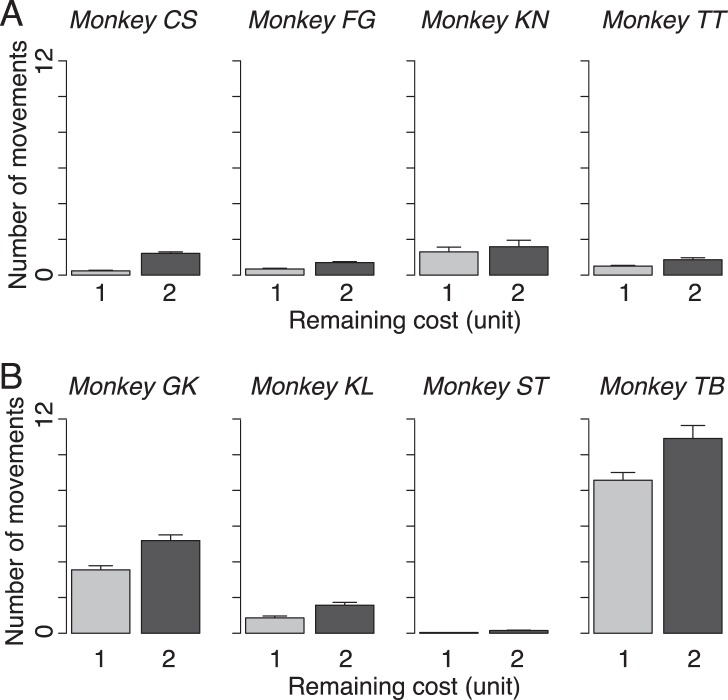
Bar touch movements during delay periods (mean ± SEM). A Effort-sensitive monkeys. **B** Effort-insensitive monkeys.

In the delay trials, the monkeys sometimes repeatedly touched and released the bar during delay periods, even though these movements would not reduce the periods. We measured the average number of bar touches during the delay period for each monkey. In the effort-sensitive monkeys, there were on average fewer than 1.5 times touches in both shorter and longer delay periods (i.e., 1 and 2 CU; Fig. 4A). Although there was no overall group effect between the effort-sensitive and effort-insensitive monkeys in bar touches during the delay (repeated measures two-way ANOVA, main effect of group, *F*_(1, 6)_ = 1.9, *p* = 0.2, Fig. 4A vs. 4B), two of 4 effort-insensitive monkeys (monkeys GK and TB) made 4 to 11 times of bar touches (Fig. 4B).

### Joint Effect of Predicted Costs and Satiation on Error Rates

**Figure 5:**
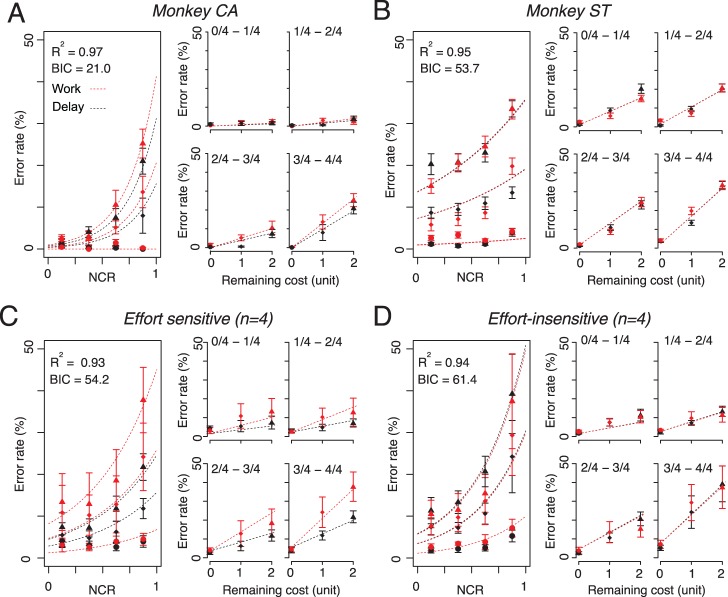
Satiation effect on error rates. Left: Error rates (mean ± SEM) as a function of normalized cumulative reward (NCR) for monkeys. Right: Error rates (mean ± SEM) as a function of remaining cost for each quartile of NCR. Red and black symbols are work and delay trials, respectively. Red and black dotted curves and lines are the best fit of Eq. 7.

The subjective value of reward should decrease as physiological drive state changes from thirst to satiation. In every daily session, the monkeys were allowed to work until they stopped by themselves, so that the data were collected as the monkeys approached satiation. Previously, we have shown that error rates of the same instrumental bar-release as used here increased monotonically as each session progressed and as the monkeys moved from thirst to satiation [9], [13]. To examine the joint effect of satiation and predicted costs on error rates, we divided each session’s data into consecutive quartiles based on normalized cumulative reward (NCR), and analysed error rates in each quadrants (see Materials and Methods). As NCR increased, the overall error rate also increased (Fig. 5). When we pick up one quadrant data (e.g., NCR 0.75*–*1, Fig. 5A right), the error rate increased as a function of remaining cost. We explained the data with joint effect of costs with satiation formulated as Eq. 7 (see Methods). For this modeling we assumed that cost factor was constant across NCRs while effect of satiation is modeled as exponential function of NCR. For both effort-sensitive and effort-insensitive monkeys, the error rates were well explained by Eq. 7 for each individual monkey (effort-sensitive, *R^2^* = 0.89±0.06, e.g., Fig. 5A; effort-insensitive, *R^2^* = 0.87±0.11, e.g., Fig. 5B), and for the average across monkeys (effort-sensitive, *R^2^* = 0.93, Fig. 5C; effort-insensitive, *R^2^* = 0.94, Fig. 5D). This result suggests that discounting factors, *k_w_* and *k_d_*, are constant as satiation level changes.

When we use equal value for *k_w_* and *k_d_* in Eq 7 to explain the error rate in effort-insensitive monkeys, the goodness-of-fit did not change (individual, *R^2^* = 0.87±0.12; average across monkeys, *R^2^* = 0.94). In these cases, the model gave smaller Bayesian Information Criterion (BIC) values (BIC_same_ = 69.7±19.5), compared with those of the model having different value for workload factors (BIC_dif_ = 72.7±19.6; *p*<0.0001; paired t-test). Thus, performance of effort-insensitive monkeys was well described by the model assuming the same discount effect between work and delay, along with satiation level changes.

## Discussion

### Isolating the Effect of Effort on the Motivation for an Instrumental Action

It is widely known that the motivation for instrumental action for rewards decreases as the delay for receiving the reward and as the workload to obtain the reward increases. Here we assessed monkeys’ performance of instrumental action either followed by an additional 1 or 2 action requirement for obtaining a reward (work trials) or followed by delay-to-reward (delay trials). We used ca. 3.6 or 7.2 s for delay duration, a time equivalent that needed to complete 1 or 2 work trials, respectively. Trial types (either work or delay) and number of remaining cost unit (0, 1, or 2) were informed by visual signal at the beginning of each trial. This design allowed us to identify the added effect from of workload over delay alone by comparing the performance between 2 trial types. All 8 monkeys showed a significant effect of delay on error rates. Half of our limited sample showed that work increased the error rates over those seen with delay only. Thus, in this group there was a dichotomy between monkeys being sensitive to effort, and those being insensitive.

This dichotomy was not consistently observed as a group effect of cost type on reaction time. Using similar instrumental tasks, we have shown that reaction times are less reliable measurement of motivation as having consistent relationship with measured parameters (e.g., reward size and delay-to-reward) than the error rates [9]. We suggest that this may be because reaction time data can only be collected in successful trials, when the monkeys have already reached the motivational threshold to act.

### Hyperbolic Discounting by Predicted Effort

As we reported earlier [9], error rates in delay trials increased linearly as predicted delay-to-reward duration increased (*cf.* Fig. 2). Because the error rates of these tasks are inversely related to motivational value, the linear effect is well modeled by hyperbolic discounting of the reward value by delay duration [9]. Here, we find that the error rates for work trials also increase linearly with predicted workload, i.e., they are also well modeled by a hyperbolic discounting of reward value by workload (*cf.* Eq. 4). Similar to what was seen for delay discounting, the work discounting was fit better by a hyperbolic function than an exponential function. Accordance with the assumption that the effect of workload is sum of effort and delay, the extra cost over delay in work trials (i.e., *k_w_–k_d_*) is regarded as cost of effort. For the 4 effort-sensitive monkeys, the subtractive effects of time from work on performance are well-modeled by linear relation, suggesting that reward value is hyperbolically discounted by predicted effort in these cases. These results are in accordance with a study using human subjects, in which the subjective value for sexual rewards is discounted hyperbolically with physical effort (i.e., the power of hand grip squeezing) as well as delay duration, when male subjects choose between a costly option for larger reward (i.e., viewing erotic picture for a long time) and a minimal cost option for smaller reward (i.e., viewing the picture for a short time) [14]. Since we used non-choice paradigm, our results support the idea that the linear discounting by cost is the properties of representation of reward value, rather than the consequence of cost-benefit comparison or decision-making process.

### Cost-discounting and Devaluation by Satiation

Motivation for instrumental action is affected by a subject’s internal state, for example, the change in motivational value according to satiation level that we have seen here and in the past. In our previous analysis, as the animal proceeds from thirst to satiation, motivational value is reduced as rehydration, an effect we modeled with sigmoidal function of cumulative reward [9], [13]. In addition, the effect of delay scales linearly, that is, it is multiplicative, the effect of satiation on motivational value; this form has the advantage that the discount factor, *k*, can be assumed to be constant and independent of satiation level [9]. Here, we extend this assumption to effort discounting, as shown by the discounting model with exponential devaluation by satiation level fits the data in both delay and work trials well in both effort-sensitive and -insensitive monkeys (*cf.* Fig. 5).

In this study the relative effect of effort and delay on motivational value appears constant across levels of satiation, suggesting that cost-discounting and devaluation are independent process. This offers the possibility that the neural signals underlying cost-discounting also scale linearly with effort and delay, making it possible to study the neural signal at any point during a session, no matter what the satiation level is. In practice it would seem best to study the neural signals in individuals that are effort sensitive.

### Sensitivity to Delay and Effort as Costs for Obtaining Reward: Traits and Possible Neural Substrates

What is the difference between the effort-sensitive and -insensitive monkeys? One possibility is that the effort-insensitive monkeys estimate smaller costs on work trials because they are individuals that respond quickly to the target and thereby receive their reward more quickly, ameliorating the effect of the extra work. We rule out this explanation because there is no systematic reaction time difference between two groups (*cf.* Fig. 3). Another possibility is that the monkey is willing to take risk in advance of making errors into account for the predicted future cost. We also exclude this possibility because there was no significant difference in the cost factors between two groups.

It is known that discounting bias depends on species. For example, common marmosets tolerate long delays but are less likely to make more effort to travel farther to obtain greater reward than cotton-top tamarins [15]. This cost estimation system may be genetically encoded, or it could arise from interaction with the environment, or most likely, it arises from the interaction of these factors. This raises the possibility that the past experiences of our monkeys had a systematic effect on their cost estimation, since some of our monkeys had been performing reward schedule task for years before this experiment. However, the effort-sensitive monkeys were not distinguishable from the effort-insensitive monkeys by their history with the reward schedule task. Impulsivity for instrumental action was indistinguishable between 2 groups; there was no systematic tendency in error patterns. Two of 4 effort-insensitive monkeys showed more bar releases during the delay (*cf*. Fig. 4). This could be a sign of a monkeys’ trait tendency to be effort-insensitive, although this does not explain all 4 insensitive ones.

From observations in animals given selective ablations and in human neuroimaging, we can suggest several neural substrates that might be responsible for the differences in relative sensitivity between effort and delay we have seen here. In rodent anterior cingulate cortex lesions affect effort-based decision making, whereas orbitofrontal cortex lesions affect delay–based decision making [5], [16]. Human fMRI studies suggest that there is an anterior cingulate-insula network that emphasizes effort discounting and a ventral striatum-ventromedial prefrontal cortex network for delay discounting [14]. There is also evidence from monkeys with ablations of different prefrontal areas that orbitofrontal cortex is essential for evaluation of reward size and delay duration, whereas lateral prefrontal cortex is essential for normal evaluation by putting these two dimensions into discounted outcome value [17]. In addition, it appears that serotonin is related to temporal discounting [18], [19], whereas mesolimbic dopamine is related to effort discounting [20].

Within the monkey population there seems to be a significant dichotomy in the sensitivity governing whether working is more costly than waiting, possibly arising from a constitutional or genetic trait.
